# Ways to investigate vestibular contributions to cognitive processes

**DOI:** 10.3389/fnint.2014.00040

**Published:** 2014-05-15

**Authors:** Antonella Palla, Bigna Lenggenhager

**Affiliations:** ^1^Department of Neurology, University Hospital ZurichZurich, Switzerland; ^2^Zurich Center for Integrative Human Physiology, Institute of Physiology, University of ZurichZurich, Switzerland

**Keywords:** caloric vestibular stimulation, galvanic vestibular stimulation, natural vestibular stimulation, cognitive neuroscience

Originally conceived as a primary system embedded into reflex generation for spinal and ocular-motor control, there is now an exciting and rapidly growing line of research showing that the vestibular system—which is intrinsically highly convergent with other sensory and motor signals (Angelaki and Cullen, 2008)—interacts with various cognitive processes such as spatial navigation (Angelaki et al., 2009), space perception (Ferre et al., 2013a), body representation (Lopez et al., 2010; Ferre et al., 2013c), mental imagery (Lenggenhager et al., 2008; Falconer and Mast, 2012; Van Elk and Blanke, 2014), attention (e.g., Figliozzi et al., 2005), memory (e.g., Smith et al., 2010), risk perception (Mckay et al., 2013), and even social cognition (Lopez et al., 2013).

Insight in this area has mostly been gained through the use of standardized clinical equipment such as caloric, galvanic, and vestibular evoked myogenic potential (VEMP) devices. While these techniques bear several advantages, it is important to recall that they stimulate the vestibular organ in an unnatural and non-physiological way. Such artificial stimulation might be exploited for specific questions, but might hamper the investigation of others. Below we will briefly describe the advantages and disadvantages of various stimulation techniques for investigating specific research questions from cognitive neuroscience (see Figure 1 for a summary). Emphasis will be put on those stimulation methods that best approximate natural movement, i.e., whole-body motion platforms. We will provide evidence that even though these highly sophisticated apparatus are technically demanding and hence not routinely available, they are indispensable for investigating certain vestibular-cognitive interactions.

**Figure 1:**
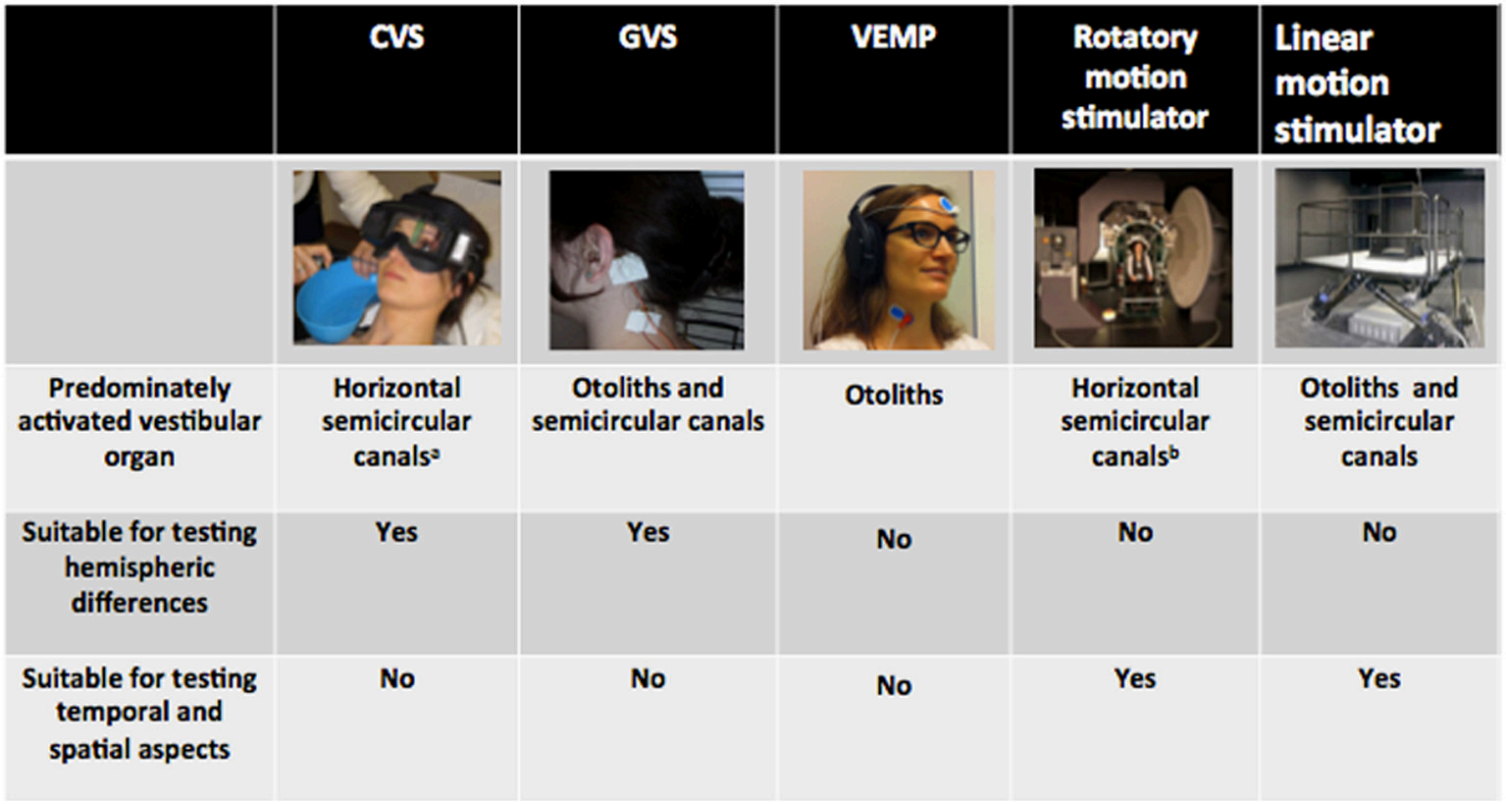
**Summary of the most common methods to stimulate the vestibular system and their suitability for testing specific cognitive research questions**. ^*a*^Typical subject position during CVS, predominantly stimulating the horizontal semicircular canals. Note that the vertical semicircular canals can also be stimulated by changing the subjects head position in order to place the vertical canals gravitationally horizontal. ^*b*^Typical subject position on a custom-built rotatory chair with the subject placed such that their center of head rotation falls along the chair rotation axis. This type of rotation predominantly activates the horizontal semicircular canals. Note that the vertical semicircular canals and the otoliths can also be activated by displacing the subject's head out of the chair rotation axis (i.e., lateral displacement or tilting).

## Ways to stimulate the vestibular system

The most frequently used techniques for investigating vestibular influence on cognitive processes are caloric (CVS) and galvanic (GVS) vestibular stimulation (for CVS see e.g., Been et al., 2007;for GVS see e.g., Utz et al., 2010). Initially developed for clinical diagnostics, they now play an increasingly important role in cognitive neuroscience, mainly due to their safe, inexpensive, non-invasive and easy applicability. In addition, they have enthusiastically been proposed as a potential therapeutic method for various bodily, affective and cognitive disturbances (e.g., Ramachandran and Mcgeoch, 2007; Preuss et al., 2014). Yet, next to some technical limitations (e.g., co-activation of other sensory systems or side effects Lenggenhager et al., 2008), some peculiarities must be considered in order to appraise their value.

*CVS* induces an endolymphatic flow within the semicircular canals by creating a temperature gradient from one end to the other end of the canal (for details about the technique, see e.g., Fetter, 2010). It is usually applied in participants placed in a supine position with their head tilted 30° forward. This way the horizontal semicircular canals are gravitationally horizontal, and thus most strongly stimulated. By changing the orientation of the head, i.e., positioning one of the vertical semicircular canals gravitationally horizontal, these latter can also be targeted. The vestibular sensation induced by CVS is complex and comprises feelings of rotation along the frontal [i.e., coronal rather than axial (i.e., yaw)] plane, floating, tilting to either side, being elevated or pulled down (e.g., Kolev, 2001). Importantly, the complexity of vestibular sensations reflects the non-physiological nature of the stimulus.

*GVS*, on the other hand, is transmitted via two electrodes placed over the mastoid process. The behavioral response induced by GVS is also complex, including sensations of rocking, pitching, tilting, and rotations. This complexity most likely originates from the simultaneous stimulation of *all* peripheral afferents (Goldberg et al., 1984) including those of the semicircular canals and otoliths (Curthoys and Macdougall, 2012). The direction of vestibular sensation can be modulated depending on the current flow, being unidirectional when using direct current flow and bidirectional when using sinusoidal flow (e.g., Stephan et al., 2005).

Another frequently used stimulation technique in clinical setting is vestibular stimulation through brief pulses of air-conducted sound or bone-conducted vibration. Specifically, vibrations applied to the head, most commonly the forehead or the mastoid, are thought to cause small linear movements of the bone while intense sound induces flow of the inner ear fluid through movements of the stapes. Both stimulations activate predominately otolith receptors, which are assessed by measuring electromyographic activity from the extraocular, via ocular VEMP, or sternoclaidomastoideus, via cervical VEMP. Yet, some authors argued for an additional semicircular contribution (Zhu et al., 2011). Notably, such stimulations have to our best knowledge not been used in cognitive research. Moreover, it is not known if and what sensations are exactly generated by VEMP stimulation, as up to know VEMPS have only been used to diagnose and confirm otolithic dysfunction (Rosengren et al., 2010).

Next to CVS, GVS, and VEMPs, motion stimulators are increasingly occupying an integral part in vestibular research. The pivotal advantage of motion platforms is that they provide complete quantitative information about the induced movement kinematics, including position, velocity, acceleration, and the dynamics. They can be programmed to approximate natural movements and the vestibular sensations generated can thus be closely related to the actual movement.

Custom-built rotatory chairs activate mainly the horizontal semicircular canals as the participant is usually sitting upright with the center of the head passing through the chair rotation axis (see also Figure 1). If the subject is positioned in supine, prone, or on the side, however, also the vertical semicircular canals can be stimulated. Otoliths can be activated when the center of the head is displaced eccentrically to the axis of chair rotation, e.g., by lateral displacement (centrifugal rotation) or by tilting (off-vertical axis rotation, OVAR). The behavioral response elicited by centrifugal rotation and OVAR is however also complex. The former induces a combined tilt-translation sensation such as being on a gondola while OVAR instead builds up a sense of as being translated along a circular trajectory (described as either feeling swayed around a cone during earth-vertical OVAR or as being translated around a circular path without the sense of turning, just like in a gondola of a Ferris wheel during earth-horizontal OVAR Vingerhoets et al., 2007). Finally, linear motion simulators equipped with six actuators allowing the motion and positioning in space following the 6° of freedom are now increasingly publicized for otolithic stimulation and already used in some laboratory. Yet, the frequency and displacement range is compared to rotatory chairs still limited. Unfortunately, such linear motion stimulators are not yet used for standard clinical testing and knowledge on vestibular induced sensations is scare.

## “What” and “when” to stimulate the vestibular system

The vestibular activation patterns and corresponding sensations as well as the technical limitations of the various vestibular stimulators might importantly influence results and conclusions of neurocognitive research. By means of some exemplary studies, in the following we aim to provide suggestions on which vestibular technique should preferentially be considered for which type of cognitive research question.

We propose that the cognitive processes influenced by vestibular signals can roughly be gathered into two groups: (a) cognitive processes in which temporal and spatial aspects are important and (b) cognitive processes in which neural hemispheric asymmetries are important.

The cognitive processes with a clear spatial component include mental space representation such as the mental number line (Hartmann et al., 2012; Ferre et al., 2013b) and mental body transformation or perspective-taking (Lenggenhager et al., 2008; Falconer and Mast, 2012; Van Elk and Blanke, 2014). Several studies have investigated whether vestibular sensations influence mental space and movement representations. In these studies, the consideration of the specific vestibular organ that is predominantly activated by the stimulator as well as the perceived direction of the induced movement is crucial. For instance, for addressing questions about *linear* mental space representation [e.g., in order to simulate motion along a number line in space (Hartmann et al., 2012)], the use of stimulators that activate the otoliths (i.e., GVS, VEMP^1^, rotational or linear motion stimulators) might be the most appropriate. We believe however, that for the above-mentioned studies, linear motion simulators (if available) should be preferred over artificial stimulation as the cognitive task and the vestibular stimulation can be matched more precisely in space and time. This is particularly true as the percept induced by artificial stimulation is very variable among participants. Corroborating this assumption, Hartmann et al. (2012) showed by means of linear motion simulators that small number generation is facilitated during leftwards and large number generation during rightwards horizontal movements, whereas Ferre et al. (2013b) did not find any spatial bias on the mental number line using GVS. The use of motion simulation allowed Hartmann and colleagues (2012) to let participants generate the numbers during the peak of linear acceleration and to demonstrate that linear mental space is plane-related as they could not replicate their findings when exposing the participants to vertical plane stimulation. Such precise motion simulation in space as well as in time is not possible with artificial stimulation.

Similarly, the choice of vestibular stimulator could be important when investigating mental own body transformations in space (Lenggenhager et al., 2008; Falconer and Mast, 2012; Van Elk and Blanke, 2014). As these tasks require participants to mentally rotate their body^2^, using stimulators that predominantly activate the semicircular canals (i.e., CVS or rotatory chairs) might be the most appropriate, since these best imitate a rotational movement. Again, differences in vestibular stimulation could presumably explain the discrepancies found between existing studies on this topic. For instance, while Lenggenhager et al. (2008) and Dilda et al. (2012) found a general and direction-unspecific slowing of mental own body rotation during GVS, Falconer and Mast (2012), using CVS, and Van Elk and Blanke (2014) using a rotatory chair reported direction-specific speeding up of mental rotation when the induced and the imagined rotation were congruent.

Whereas the great value of motion simulators consists in the ability to explore temporal and spatial processes of cognition and might thus be very important for future research investigating embodied and spatial cognition, they may be inferior as compared to CVS and GVS for the investigation of strongly lateralized (i.e., right or left cerebral hemispheric) cognitive aspects such as risk perception (Mckay et al., 2013) or emotion (Preuss et al., 2014). Vestibular stimulation has been showed to activate a large neural network, centered in multisensory areas of the temporo-parietal cortex and posterior insula (see e.g., Lopez and Blanke, 2011 for a recent review). Co-activation of shared neural networks might make vestibular stimulation an interesting tool for the manipulation of various cognitive tasks, similar to cortical stimulation techniques (e.g., tDCS or TMS). For this line of research, we would advise the use of CVS and/or GVS for two main reasons: First, both techniques revealed a hemispheric dominance of vestibular projections (i.e., stimulate preferentially one hemisphere depending on the stimulation pattern), making them interesting for testing interference with hemispheric specialization (Dieterich et al., 2003). Related to self-risk estimation and the hypothesis of asymmetrical brain processing, one could for example, expect stronger results with CVS than with rotatory motion stimulator, as the former activates cortical hemispheres more asymmetrically than the latter (for a CVS study see Mckay et al., 2013). Second, as for these studies, it is usually interesting to know which exact brain mechanism underlie the interaction, a further advantage of CVS and GVS is that they can easily be used in a scanner. While advantages in more mobile neuroimaging techniques (e.g., NIRS) might soon overcome this limitation, up to now there are no neuroimaging studies using real motion simulation.

## Conclusion

In sum, artificial galvanic and caloric vestibular stimulation techniques are very useful and to date constitute a keystone in cognitive neuroscience. We argue however, that for certain specific research questions—mainly those assessing vestibular influences on spatial cognition—the use of more sophisticated motor stimulators is indispensable. Generally, we believe that the complexity of the vestibular system and the growing repertory of stimulation devices (and parameters) make a critical selection of the vestibular stimulation important. This paper aims to sensitize readers from the cognitive or from the vestibular domain to these aspects when designing an experiment and interpreting the data. We also hope that future papers in this field will more often include the underlying reasoning for choosing one over another technique.

### Conflict of interest statement

The authors declare that the research was conducted in the absence of any commercial or financial relationships that could be construed as a potential conflict of interest.

## Footnotes

^1^Even if otolith stimulation through VEMPS do presumably not evoke any self-motion perception, it might be interesting to investigate its implicit influence on certain cognitive tasks.

^2^This is of course not the case when the mental transformation is linear, e.g., when simulating moving forward or backwar.
